# DDCATNet: Effective Deep Learning-Based Illumination Color Cast Estimation Approach for Achieving Computational Color Constancy

**DOI:** 10.3390/s26113313

**Published:** 2026-05-23

**Authors:** Ho-Hyoung Choi

**Affiliations:** School of Dentistry, Advanced Dental Device Development Institute, Kyungpook National University, Jung-gu, Daegu 41940, Republic of Korea; chhman2000@msn.com; Tel.: +82-010-6771-6680

**Keywords:** HVP-based CCC, DCNN, channel-wise and spatial attention-based DDCATNet, dense dual connection (DDC), aggregate transform, illuminant estimation

## Abstract

Digital camera sensors are designed to capture a wide range of incident illuminants, enabling the creation of high-quality images. However, these sensors lack the capability to differentiate between the color of the source illuminant and the actual color (or original color) of the object being captured. For this reason, the computational color constancy (CCC) was introduced and has been developed over decades. The CCC is an approach to modeling the color perception of the human visual system (HVS) by ensuring accurate object color determination under varying source illuminant conditions. At the core of human visual perception (HVP)-based CCC is attaining higher accuracy in scene illuminant estimation. The emergence of deep convolutional neural networks (DCNNs) was a recent innovation in accurate illuminant estimation, fundamentally transforming the CCC research landscape. Nevertheless, accurate illuminant estimation still remains a huge challenge for both traditional and state-of-the-art (SOTA) approaches. To further advance precision in illuminant estimation, this article presents a novel learning-based illumination color cast estimation approach to HVP-based CCC. Most importantly, the proposed approach is intended to integrate informative features into both channel and spatial regions while preserving long-term dependency feature information with the use of dense skip connections. To achieve these objectives, the proposed Dense Dual Connection Aggregated Transform Network (DDCATNet) architecture is designed to comprise several modules: shallow feature extraction, channel-wise and spatial feature-based Dense Dual Connection (DDC), fusion of the dense channel-wise attention (CA) and spatial attention (SA) branches through a gate mechanism (GM) unit, and aggregate transform. It is worth noting that both the CA blocks and the SA blocks in the DDC module are characterized by dense and cascading connections, meant to preserve long-term feature information and modulate different-level feature information at both global and local scales. The densely connected CA branch (DCA) and the densely connected SA branch (DSA) are also highly effective in securing high-contribution information while suppressing redundant data. The GM unit is integrated at the back of the DDC module, fusing the two DCA and DSA branches to ensure the adaptive merging of useful hierarchical feature information and the extraction of more valuable feature information. As a result, the proposed DDCATNet architecture significantly enhanced precision in illuminant estimation, thereby improving performance. In rigorous experiments on a wide range of datasets, the proposed DDCATNet approach outperformed its SOTA counterparts, validating the efficacy and generalization capabilities, as well as robust camera-invariance, across diverse, single- and multi-illuminant datasets and model architectures.

## 1. Introduction

The computational color constancy (CCC) is an approach to modeling the color perception of the HVS by ensuring accurate object color determination under varying source illuminant conditions. The HVP-based CCC approaches represent a technology for estimating and removing the source illuminant color of the captured image, and thereby ensuring consistency in color perception across different lighting conditions. Consistent color recognition spans a broad range of applications, from digital photography to automatic image processing. Traditional CCC approaches, including GW [1], GE [2], and SoG [3], were built on the statistics-based gray-world assumption concerning the color distribution of the captured image. They had the advantage of low computational costs, but their limitations were ambiguous color distribution and the resultant color distortion [4]. Other statistics-based approaches were proposed and made substantial progress in performance, including BP [5] and GI [4], based on the gray-world assumption. However, the statistical approaches presented a drawback when the requirements of the assumption—the average of all colors in a captured image is gray—were unmet, resulting in a biased or misleading inference.

To address limitations in traditional approaches, researchers have been proposing learning-based methods. The learning-based approach is in distinct contrast to their statistic-based counterparts, by using a huge amount of training datasets to estimate the source illuminant color. The learning-based approach was a major innovation in the CCC and has fundamentally transformed the research landscape, leading to significant accuracy improvements. The early learning-based models were characterized by the introduction of gamut mapping, as referenced in [6,7]. Another wave of innovation was driven by advancements in machine learning, as in the regression model [8] and the CCC approach [9]. Learning-based models have achieved considerable performance improvements but struggle when handling image noise, given that the models are designed to estimate the source illuminant color by performing spatial domain data classification. To avoid this limitation, a new learning-based approach, the frequency domain transformation method, as in FFCC [10], was proposed by shifting from the spatial domain to the frequency domain. Most recently, DCNNs represent a significant turning point in learning-based CCC approaches. Notable models include C4 [11] and FC4 [12]. In particular, the latter is characterized by focusing on confidence-spatial regions in illuminant estimation, employing a confidence-pooling approach to enable the automatic identification of confidence-spatial regions. Notwithstanding ongoing advancements, a critical challenge persists for learning-based CCC approaches, attributed to variations in spectral sensitivity [13,14]. To counter the challenge, research has continued to evolve, coming up with innovative proposals. For instance, IGTN [15] adopts a metric learning approach with training focused solely on scene-independent illuminant color features. Quasi-unsupervised approaches use semantic feature maps of achromatic color representations of objects to achieve enhanced cross-sensor generalization. Other researchers have attempted to tackle the multi-sensor challenge by presenting domain adaptation methodologies in [16,17], as well as suggesting training the model to acquire a device-independent intermediate representation [13]. CS [18] introduces an innovative approach that uses multi-unlabeled images for illuminant estimation. As a step forward from the CS approach, CLCC [19] applies contrastive learning to ensure that the same scene images captured under distinct source illuminants exhibit distinct representations. In the context of the advancements in the CCC, this article presents a novel learning-based illumination color cast estimation approach to HVP-based CCC. Most importantly, the proposed approach is intended to integrate informative features into both channel-wise and spatial regions while preserving long-term dependency feature information with the use of dense skip connections. To achieve these objectives, the proposed Dense Dual Connection Aggregated Transform Network (DDCATNet) architecture is designed to comprise several modules: shallow feature extraction, channel-wise and spatial feature-based Dense Dual Connection (DDC), fusion of the dense channel-wise attention (CA) and spatial attention (SA) branches using a gate mechanism (GM) unit, and aggregate transform. In the DDC module, both the CA blocks and the SA blocks are characterized by dense connectivity and cascading flow, meant to preserve long-term feature information and modulate different-level feature information at both global and local scales. The two densely connected branches are also highly effective in securing high-contribution information while suppressing redundant data. The GM unit, based on a 1 × 1 convolutional layer, is integrated at the back of the DDC module, fusing the two dense branches to ensure the adaptive merging of useful hierarchical feature information and the extraction of more valuable feature information. As a result, the proposed DDCATNet architecture significantly enhanced precision in illuminant estimation, thereby improving performance.

The key contributions of this article are summarized below:

♦ The proposed DDCATNet introduces a novel concept of integrating the CA and SA mechanisms into a DDC module. This integration enables the adaptive recalibration of global and local feature responses by explicitly modeling the interdependencies between channel-wise and spatial features.
♦ The DDC module is specifically designed to modulate multiple-level features. Additionally, the GM unit is used to fuse hierarchical features, ensuring the preservation of significant information. The DCA and DSA, characterized by dense connectivity and cascading flow, are designed to effectively capture diverse attention and secure high-contribution information. The GM unit contributes to adaptively distilling more useful feature information across both short-term and long-term dependencies.
♦ The proposed DDCATNet architecture made innovative progress in illuminant estimation accuracy. In the proposed architecture, the DDC module has significantly improved the representational and discriminative learning capabilities, and its densely connected structure facilitates the full exploitation of multiple-level information and ensures the optimal information flow across modules.

The remaining sections of this article are structured as follows: Section 2 provides a comprehensive exposition of the proposed DDCATNet architecture, Section 3 discusses the experimental findings and evaluations, and Section 4 summarizes the key discussions.

## 2. Approach

Variations in illuminant conditions significantly affect the performance of downstream tasks [20,21]. The primary objective of CCC is to estimate the scene illuminant and compensate for the color cast induced by non-canonical illumination. The majority of CCC approaches are designed to perform scene illuminant estimation and correction. Although decades of research and development have advanced the field of CCC, both significant potential and challenges remain for improving illuminant estimation accuracy. In this context, the proposed DDCATNet architecture is primarily intended to achieve higher precision in illuminant estimation, thereby improving performance.

**Figure 1:**
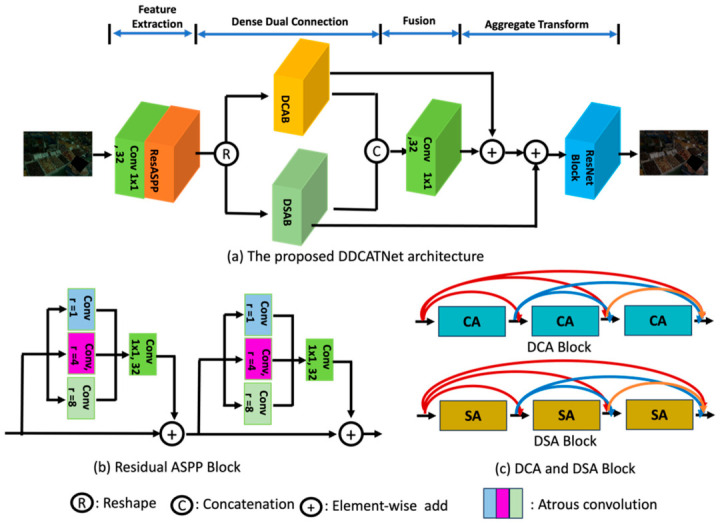
The proposed DDCATNet architecture.

As illustrated in Figure 1, the proposed DDCATNet architecture comprises shallow feature extraction, channel-wise and spatial feature-based Dense Dual Connection (DDC), fusion of the dense channel-wise attention (CA) and spatial attention (SA) branches using a gate mechanism (GM) unit, and an aggregate transform. During the first phase, called feature extraction, the Residual Atrous Spatial Pyramid Pooling (RASPP) Block offers several distinct advantages. The RASPP Block effectively extracts rich hierarchical feature information and mitigates the overfitting problem. To address global and local variations in illuminance, the DCA and DSA, characterized by dense connectivity and cascading flow, are designed to effectively mitigate the long-term dependency problem. The two densely connected branches are primarily intended to preserve long-term feature information and modulate different-level feature information at global and local scales. The proposed DDCATNet architecture has integrated the two dense branches into the DDC module to enhance the representation capability. This integration significantly improves illuminant estimation accuracy, positioning the proposed DDCATNet as superior to its DCNN counterparts. This section provides a comprehensive overview of the proposed DDCATNet architecture.

### 2.1. Overview of the Proposed DDCATNet Architecture

A DCNN-based architecture typically adopts one of two approaches to achieving CCC. One is directly estimating the white-balancing image, and the other is removing illuminant color cast by estimating an illuminant map that represents the illumination value at each pixel in the given image. The first approach outputs white-balanced images that reflect variations present in the input images, and the second approach outputs corrected images by removing the illuminant color. The first approach involves defining a mapping function f(·) and inputting the image $I\left(x,\;y\right)\in R^{H\times W\times 3}$. The resulting output, $E\left(x,\;y\right)=f(I\left(x,\;y\right))$, represents the illuminant color map, where $E\left(x,\;y\right)\in R^{H\times W\times 3}$. The second approach corrects the illuminant of the input image by utilizing the function E(x,y). To estimate the illuminant color map, the second approach extracts shallow features, defined as a function $F_{s}$, from the input image, using the shallow feature extraction module, described as follows:

(1) $$F_{S}=H_{SF}(I(x,\;y)),$$

where $F_{S}\in R^{H\times W\times 3}$ denotes the extracted shallow feature, while $H_{FS}$ is defined as a shallow feature extractor.

Based on Equation (1), the resulting shallow features are fed into the dense CA and branches. That is:

(2) $$F_{CA,k}=H_{CA}(U_{CA}),$$

(3) $$F_{SA,k}=H_{SA}(U_{SA}),$$

where $H_{CA}$ and $H_{SA}$ refer to the CA blocks and the SA blocks, respectively. $U_{CA}$ and $U_{SA}$ denote the inputs to the CA blocks and the SA blocks, respectively. The results from the two dense branches are described as follows:

(4) $$F_{DCA}=F_{CA,0}+F_{CA,1}+F_{CA,2},$$

(5) $$F_{DSA}=F_{SA,0}+F_{SA,1}+F_{SA,2},$$

where $F_{DCA}$ and $F_{DSA}$ represent the dense channel attention (DCA) branch and the dense spatial attention (DSA) branch, respectively. In this work, k = 3, but in some cases, it can be expanded to k = 4 or k = 5 in Equations (2) and (3). The $F_{DCA}$ and $F_{DSA}$ are fused, using the GM unit, described as follows:

(6) $$F_{fu}=\;H_{gate}\left(\left[F_{DCA},F_{DSA}\right]\right),$$

where the $H_{gate}$ defines the GM unit. As depicted in Figure 1, a 1 × 1 convolutional layer is employed to merge the $F_{DCA}$ and $F_{DSA}$. $\left[\cdot \right]$ refers to the concatenation operator.

Finally, the extracted deep feature maps are fed into the aggregate transform module. This module serves as a bottleneck residual module, comprising a sequence of three stacked convolutional layers: the 1×1 Conv−3×3 Conv−1×1 Conv architecture. A skip connection is incorporated to facilitate information flow and enhance module performance [22,23,24].

### 2.2. Shallow Feature Extraction

To achieve accurate correspondence estimation, it is essential to incorporate comprehensive context information into feature representation [25]. Therefore, a large receptive field (RF) and multiple-scale feature learning are essential for obtaining a discriminative feature. The RASPP Block in Figure 1b is employed to extract discriminative features with rich context feature information. As per references [26,27,28], the ASPP module offers the advantages of extracting hierarchical feature information and expanding the RF. For the shallow feature extraction module, called feature extraction in Figure 1, the proposed architecture uses a 1×1 convolutional layer to extract the initial features. The initial features are subsequently fed into the Residual ASPP Block that generates multiple features, as in Figure 1b. The RASPP Block has three atrous convolutions with the atrous rates of 1, 4, and 8, which combine to form the ASPP Group, meaning that three atrous convolutions are cascaded in a residual manner. Accordingly, the RASPP Block expands the RFs in size, as well as enriches the architecture with diverse convolutions. The highly discriminative feature trained through the RASPP Block provides significant advantages to overall CCC performance.

### 2.3. CA and SA Blocks

A DCNN generally generates diverse feature maps across channel-wise and spatial regions, whereby each map makes a distinct contribution to the recovery of high-frequency details. Assuming that the sensitivity of the DCNN can be enhanced to capture higher-order feature information and incorporate training-relevant features, the representational power of the DCNN will be improved, thereby leading to an enhanced estimation accuracy. In this context, the CA and SA blocks are adopted for the proposed architecture to enhance the representational power, as well as improve illuminant estimation accuracy. These blocks leverage the interdependencies between channel-wise and spatial regions of the features to enhance the DCNN’s ability to capture and utilize relevant feature information effectively.

#### 2.3.1. CA Block

**Figure 2:**
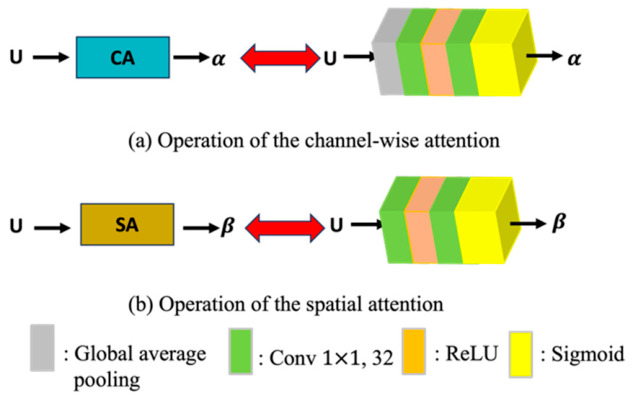
The operational sequence of the CA and SA blocks depicted in Figure 1c.

The key objective of the CA blocks is to achieve global feature recalibration. During this process, per-channel summary statistics are computed and subsequently utilized to select useful feature information maps while suppressing redundant and irrelevant feature information maps. Figure 2a provides an illustration of the architectural design of the CA blocks. Let $U=\left[u_{0},u_{1,}\;u_{2},\cdot \cdot \cdot \cdot \cdot ,u_{C}\right]$ be the input to the CA blocks. The input comprises C feature information maps with a spatial dimension of H×W. In order to produce channel-wise (CW) summary statistics $z\in R^{C\times 1\times 1}$, the global average pooling (GAP) is applied to each feature channel (FC) across the spatial dimension of H×W as in [29]. Let $u_{c}(i,\;j)$ represent the input value at a location (i, j) of the c−th channel, $u_{c}$. The c−th element of z is calculated as follows [30]:

(7) $$z_{c}=\;\frac{1}{H\times W}\sum_{i=1}^{H}\sum_{j=1}^{W}u_{c}(i,\;j),$$

Then, the CW summary statistics are applied to the GM unit with a sigmoid activation function to ensure the allocation of diverse attentions to diverse feature information maps. Let $\sigma \left(\cdot \right)$ and $\delta \left(\cdot \right)$ denote the sigmoid and ReLU [31] activation functions, respectively, and ∗ denote the convolutional operator. Let $W_{CA}^{1}\in R^{\frac{C}{r}\times C\times 1\times 1}$ and $b_{CA}^{1}\in R^{\frac{C}{r}}$ represent the weight and bias, respectively, for the first convolutional layer. This layer is followed by the ReLU activation function and employs the reduction ratio r, thereby reducing the number of channels for z. This process is reflected in the formula below:

(8) $$\alpha =\;\sigma \left(W_{CA}^{2}*\delta \left(W_{CA}^{1}*z+b_{CA}^{1}\right)+b_{CA}^{2}\right),$$

Meanwhile, the original number of channels is restored by employing another convolutional layer with the parameters $W_{CA}^{2}\in R^{C\times \frac{C}{r}\times 1\times 1}$ and $b_{CA}^{2}\in R^{C}$. CA weights, denoted as $\alpha \in R^{C\times 1\times 1}$, are mapped to the [0, 1] range through the sigmoid activation function $\sigma \left(\cdot \right)$, and then used to modify the scale of the input feature, formulated below:

(9) $$U_{CA}=\;\Phi_{CA}\left(U\right)=\;f_{CA}\left(U,\alpha \right),$$

In the context of the CW multiplication operation, the function $f_{CA}\left(\cdot \right)$ is utilized to represent the multiplication of FCs and their corresponding applicable channel weights. The function $U_{CA}$ refers to the CW recalibrated result and the function $\Phi_{CA}\left(\cdot \right)$ denotes the CA blocks.

Following the aforementioned process, the CA blocks are able to adaptively modulate the CW features in accordance with the statistical characteristics of the input information. This adaptive modulation enables the DCNN to enhance the discriminability of the CW features.

#### 2.3.2. SA Block

The global average pooling (GAP) used in the CA block serves to compress global spatial feature information into a channel statistical descriptor. However, this process entails the elimination of spatial feature information from each feature information map. In contrast, feature information, encompassing inputs and feature maps, demonstrates regional spatial variations. For instance, regions such as the edge and texture typically exhibit a higher concentration of high-frequency feature information. Conversely, the smooth regions possess a greater abundance of low-frequency feature information. Therefore, recovering high-frequency details is essential for enabling the DCNN to develop the discriminative capacity for various local regions. Here, a complementary attention mechanism referred to as SA is employed to enhance the representation capabilities of the DCNN. The key benefit of the SA block lies in the ability to prioritize attention to diverse local regions where high-frequency feature information is concentrated, and such information is of high significance but challenging to reconstruct. As shown in Figure 2b, let $U=\left[u_{0},u_{1,}\;u_{2},\cdot \cdot \cdot \cdot \cdot ,u_{C}\right]$ be the input to the SA blocks. The input comprises C feature information maps with a dimension of H×W. To facilitate the utilization of the computational neuroscience models in [32], which are based on input interdependency and motivated by local computations, a two-layer CNN is employed in conjunction with a sigmoid activation function to create an SA mask, denoted as $\beta \in R^{1\times H\times W}$. In the context of Equation (8), let $\sigma \left(\cdot \right)$ be the sigmoid activation function, and let $\delta \left(\cdot \right)$ and * signify the ReLU activation function and the convolutional operation, respectively. The SA mask is formulated as follows:

(10) $$\beta =\;\sigma \left(W_{SA}^{2}*\delta \left(W_{SA}^{1}*U+b_{SA}^{1}\right)+b_{SA}^{2}\right),$$

In Equation (10), the initial convolutional layer, parameterized as $W_{SA}^{1}\in R^{\gamma C\times C\times 1\times 1}$ and $b_{SA}^{1}\in R^{\gamma C}$, generates per-channel attentive maps. These maps are integrated into a comprehensive attentive map through the application of the subsequent 1 × 1 convolutional layer, parameterized as $W_{SA}^{2}$ and $b_{SA}^{2}$. The attentive map is normalized to a range of [0, 1] by using the sigmoid activation function. This normalization is intended to make the SA soft mask β. Let $f_{SA}\left(\cdot \right)$ be the element-wise multiplication operation on the spatial region of each feature information map and their corresponding applicable SA weights, and the function $\Phi_{SA}\left(\cdot \right)$ serve as a representation of the SA model. The spatial modulation process of input feature information with the use of β is illustrated as follows:

(11) $$U_{SA}=\;\Phi_{SA}\left(U\right)=\;f_{SA}\left(U,\beta \right),$$

During this process, the input feature information undergoes spatial modulation using the SA soft mask. The SA blocks adaptively modulate local features, which can be interplayed with global CW modulation to enhance the representation power of the DCNN.

## 3. Experimental Findings and Evaluations

This section presents a comprehensive evaluation of the experiments, validating the efficacy and generalization capabilities of the proposed DDCATNet architecture across a wide range of datasets and model architectures. The experiments were conducted using standard datasets that encompass both single- and multi-illuminant datasets. Notably, the Large-Scale Multi-Illuminant (LSMI) dataset [33] contains 7486 images captured by three distinct cameras: Samsung Galaxy Note 20 Ultra (Samsung Electronics, Suwon, Republic of Korea), Nikon D810 with Nikon24-70vr lens, and Sony α9 (ILCE-9) with SEL24105G lens. The captured scenes were acquired in natural configurations, featuring single- or multi-illuminant conditions, including both natural sunlight and artificial lighting. Table 1, sourced from reference [33], presents the composition of the multi-illuminant subsets in the LSMI dataset. The proposed DDCATNet architecture was also experimented with and evaluated, using single-illuminant datasets: the Re-Processed Color Checker dataset [34] and the Cube+ dataset [35]. The Re-Processed Color Checker dataset contains 568 images captured by two cameras, Canon 1D and Canon 5D, and the Cube+ dataset contains 1707 images. Both datasets cover a wide spectrum of real-world indoor and outdoor images. For the experiments, the proposed DDCATNet architecture was implemented, using TensorFlow [36] on an NVIDIA TITAN RTX 24G GPU.

**Figure 3:**
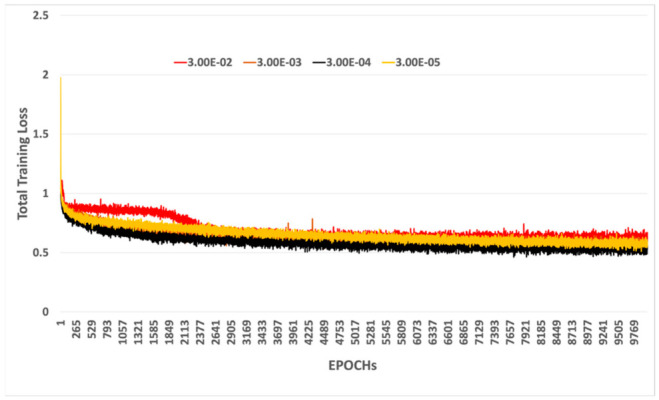
Experimental comparison of initial training rates in terms of training loss in the logarithmic space.

Figure 3 shows an experimental comparison of initial training rates, indicating that an initial training rate of 3.00 × 10^−4^ resulted in the minimum total training loss. In the figure, the *x*-axis represents epochs and the *y*-axis represents total training loss. The experiment compared four different initial training rates in terms of total training loss in the logarithmic space, which rightly demonstrates converging behaviors. The proposed DDCATNet architecture was trained for 1.2 days, totaling 10,000 epochs; the total training loss, optimized by the Adam algorithm [37], was recorded at the end of each epoch.

To complete optimal parameter configuration, empirical experiments were conducted to determine the optimal global weight decay and momentum, showing that a global weight decay of 5.7 × 10^−6^ and a momentum of 9.5 × 10^−1^ resulted in the minimum angular errors.

**Figure 4:**
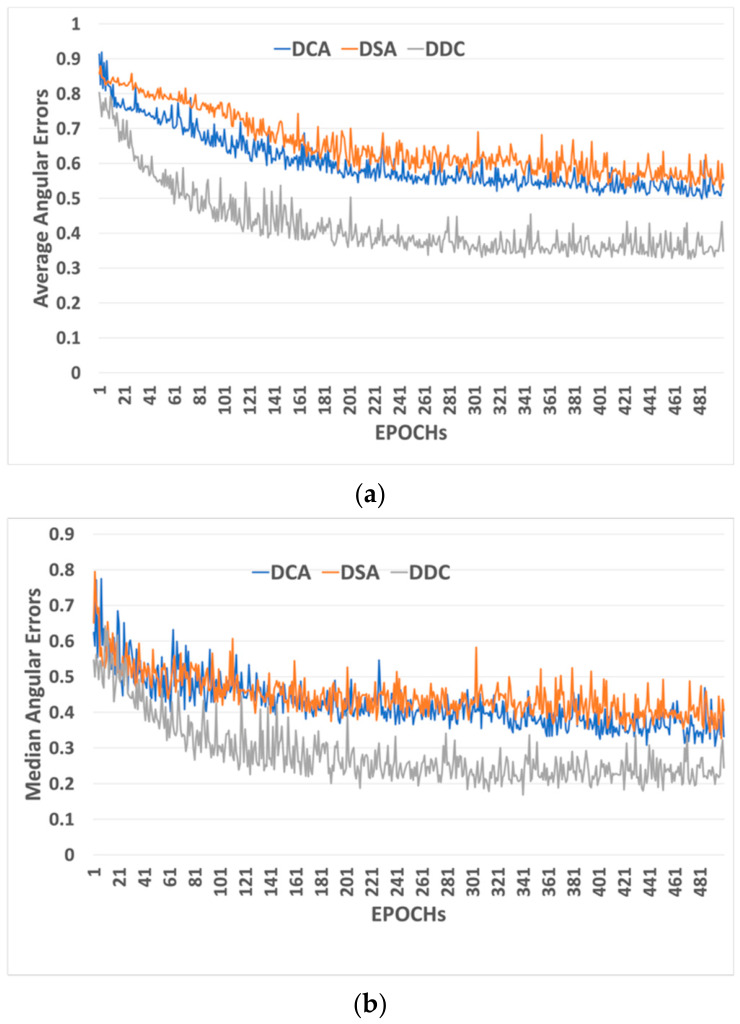
Experimental comparison of DCA, DSA, and DDC in terms of (**a**) average angular errors and (**b**) median angular errors in the logarithmic space.

**Figure 5:**
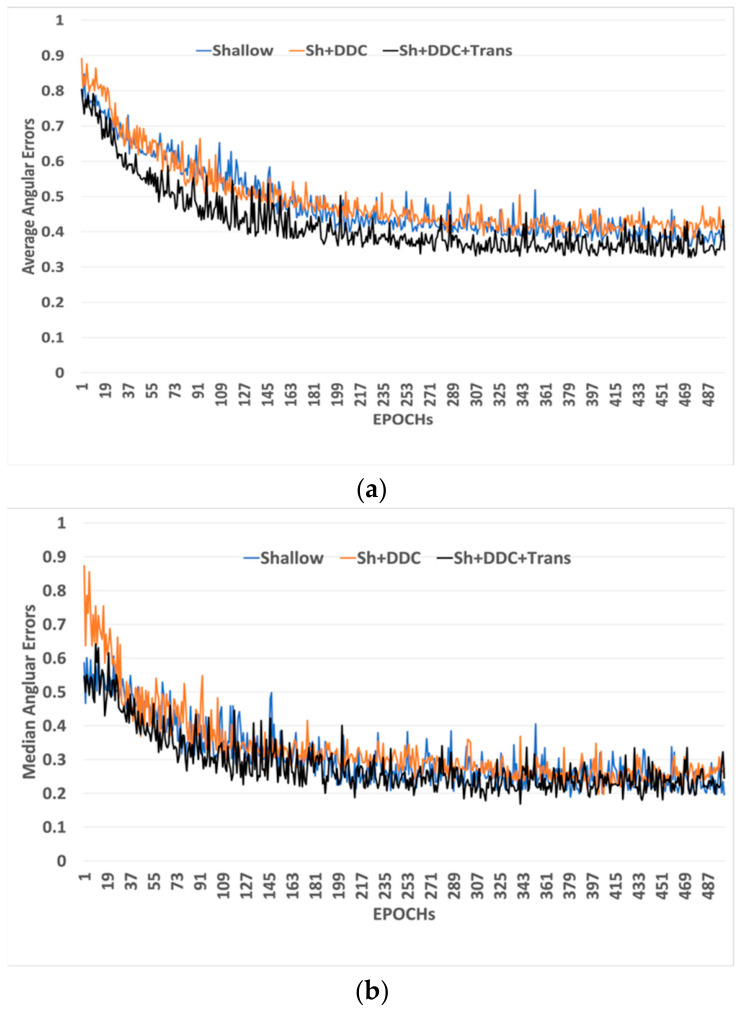
Experimental comparison of shallow, Sh+DDC, and Sh+DDC+Trans in terms of (**a**) average angular errors and (**b**) median angular errors in the logarithmic space.

Figure 4 and Figure 5 show the results of ablation studies to demonstrate the contribution of individual components, validating their interdependence to enhance the performance of the proposed DDCATNet architecture. The proposed architecture was trained for 1.2 days, totaling 10,000 epochs; the average and median angular errors, the evaluation metrics used in the experiments, were recorded at intervals of every 20 epochs.

Figure 4 compares the proposed DDC module against two simplified variants. When either DSA or DCA was removed, marked as “DCA” or “DSA”, respectively, the proposed architecture resulted in a significant increase in both average and median angular errors. It is worth noting that the DSA contributes marginally more to the performance enhancement than the DCA, primarily attributed to the high-frequency recovery, the key benefit of the SA block. Integrating both DSA and DCA, marked as “DDC”, outperformed both simplified variants, resulting in the lowest average and median angular errors. The experimental results demonstrate the contribution of all components, validating their interdependence to enhance the performance of the proposed DDCATNet architecture. Interestingly, the interdependence of all components was more pronounced in average angular errors than in median angular errors.

Figure 5 compares the proposed architecture against two simplified variants. When the proposed architecture used only the shallow feature extraction module, marked as “shallow”, it resulted in an increase in both average and median angular errors. When the proposed architecture used the shallow extraction module plus the DDC module, marked as “Sh+DDC”, it resulted in an increase in both average and median angular errors, but delivered slightly more stable performance, compared to the shallow; the DDC is able to adaptively distill more useful feature information, thereby enhancing the representational capability of the proposed architecture. When integrating all modules, marked as “Sh+DDC+Trans”, the proposed architecture resulted in the lowest average and median angular errors, outperforming both shallow and Sh+DDC. The experimental results exhibit the contribution of all modules, validating their interdependence to enhance architecture performance. Interestingly, the interdependence of all modules was more pronounced in average angular errors than in median angular errors.

The angular error measurement, used for experimental evaluations, is calculated as follows:

(12) $$e_{ANG}=arccos\left(\frac{\rho_{w}^{T}{\hat{\rho }}_{w}}{\left\|\rho_{w}\right\|\left\|{\hat{\rho }}_{w}\right\|}\right),$$

The angular error metric [38] quantifies the difference between the estimated illuminant $(\rho_{w}$) and the measured ground truth illuminant (${\hat{\rho }}_{w}$).

**Figure 6:**
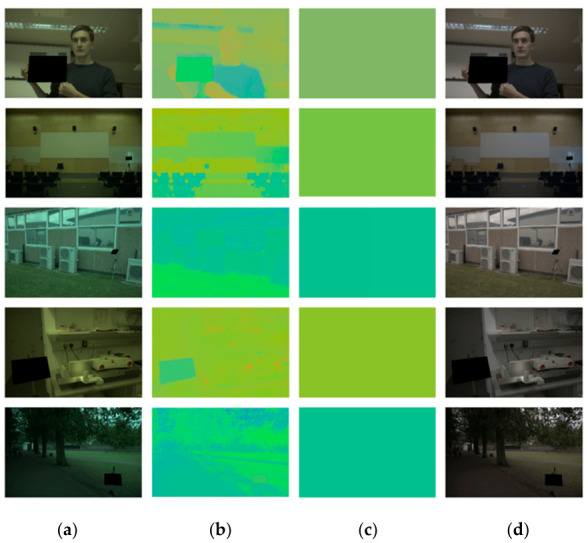
Experiment outputs with the Re-Processed Color Checker dataset: (**a**) the original images, (**b**) their paired estimated illuminant images, (**c**) the ground truth images, and (**d**) the corrected images after estimated illuminant removal.

Figure 6 shows the CCC process flow and its paired outputs: (a) the original images captured under a single fluorescence illuminant, (b) their paired illuminant maps, (c) the ground truth images, and (d) the rendered images from implementing the proposed DDCATNet architecture, resulting from experimenting with the second approach introduced in Section 2.1, Overview of the Proposed DDCATNet Architecture. The experiment used the Re-Processed Color Checker dataset, a single-illuminant dataset, to ascertain the generalization capabilities of the proposed architecture.

Table 2, Table 3, Table 4 and Table 5 provide experimental comparison summaries, demonstrating that the proposed DDCATNet architecture considerably exceeds its SOTA counterparts. The experiments used a wide range of datasets to compare the proposed architecture with its multiple SOTA counterparts, validating the efficacy and generalization capabilities across diverse single- and multi-illuminant datasets and model architectures. The evaluation metrics used for the comparative assessment are the angular errors, including the mean, median, trimean, best 25%, and worst 25%.

Table 2 presents an experimental comparison, using a single-illuminant color checker dataset, the Re-Processed Color Checker dataset. The experimental results demonstrate that the proposed DDCATNet architecture considerably exceeds its SOTA counterparts across all angular error metrics. Table 3 presents an experimental comparison, using a public Cube + single-illuminant dataset. The experimental results demonstrate that the proposed DDCATNet architecture considerably exceeds its SOTA counterparts across all angular error metrics, validating its robust scene adaptability.

Table 4 presents an experimental comparison, using the LSMI [33] and Cross-Camera datasets. Different cameras have unique sensor spectral sensitivities, leading to different color renderings. The LSMI dataset is divided into three distinct subsets based on the cameras used to capture the images: Galaxy, Nikon, and Sony cameras. On the other hand, the Cross-Camera dataset is characterized by an aggregated dataset from several cameras. The experimental results demonstrate that the proposed DDCATNet architecture considerably exceeds its SOTA counterparts across all angular error metrics, validating the effectiveness and robust camera-invariance. Table 5 presents an experimental comparison, using the LSMI and Cross-Camera datasets, divided into single-illuminant and multi-illuminant subsets. The experimental results demonstrate that the proposed DDCATNet architecture considerably exceeds its SOTA counterparts across all angular error metrics, validating the efficacy and robustness across both single- and multi-illuminant conditions. It is worth noting that while both scenes involve pixel-level illuminant estimation, the performance with the images acquired under a single-illuminant condition consistently exceeds the performance with the images acquired under a multi-illuminant condition. This signifies the complexity and challenge in the illuminant estimation and associated spatial relationships, as the number of illuminants increases.

## 4. Conclusions

The computational color constancy (CCC) is an approach to modeling the color perception of the HVS by ensuring accurate object color determination under varying source illuminant conditions. To further advance precision in illuminant estimation, this article presents a novel learning-based illumination color cast estimation approach to HVP-based CCC, named the Dense Dual Connection Aggregated Transform Network (DDCANet) architecture. The proposed architecture is composed of shallow feature extraction, channel-wise and spatial feature-based Dense Dual Connection (DDC), fusion of the dense channel-wise attention (CA) and spatial attention (SA) branches using a gate mechanism (GM) unit, and an aggregate transform. The novel integration of components and modules significantly differentiates the proposed architecture from its SOTA counterparts by deepening the architecture and enhancing both representational and discriminative learning capabilities. In the shallow feature extraction module, the Residual ASPP block modulates multiple-level global features. The DDC module is designed to preserve long-term feature information and modulate multiple-level feature information at both global and local scales. Specifically, the DDC comprises the DSA and DCA, characterized by dense connectivity and cascading flow. The two dense branches are highly effective in securing high-contribution information while suppressing redundant data. During the fusion, the GM unit, integrated at the back of the DDC, is used to merge the two dense branches, thereby adaptively merging useful hierarchical feature information and extracting more valuable feature information. Figure 3 summarizes the result of hyperparameter optimization experiments, enhancing the performance of the proposed DDCATNet architecture. Figure 4 and Figure 5 summarize the ablation experiments to demonstrate the contribution of all components, validating their interdependence, to enhancing overall performance. Table 2, Table 3, Table 4 and Table 5 present the comparative experiment summaries. The results indicate that the proposed DDCATNet architecture substantially outperformed its SOTA counterparts in rigorous comparative experiments on a wide range of datasets, validating the efficacy and generalization capabilities across diverse, single- and multi-illuminant datasets and model architectures.

In conclusion, the proposed DDCATNet architecture holds promising potential for areas and applications that demand camera- and illuminant-invariance.

## Figures and Tables

**Table 1:** Composition of the multi-illuminant subsets in the LSMI dataset [33].

Camera	2-Illuminant Scenes	3-Illuminant Scenes	Total Scenes	Total Images
Samsung Galaxy Note 20 Ultra	1000	125	1125	2500
Nikon D810	916	39	955	1988
Sony α9	1135	182	1317	2998

**Table 2:** Experimental comparison of the proposed DDCATNet architecture (“Ours”) and its SOTA counterparts using the single-illuminant Color Checker dataset [34].

Method(s)	Mean	Median	Tri-mean	Best 25%	Worst 25%
Patch CNN [39]	2.63	1.98	-	-	-
AngularGAN [40]	6.09	2.59	-	-	-
CyscleGAN [41]	3.40	2.60	2.80	0.70	7.30
LSMI-U [33]	2.15	1.56	1.70	0.54	4.68
AWB [42]	12.19	11.40	11.87	9.35	15.75
ESE [43]	2.34	1.84	1.94	0.64	4.94
CDNet [44]	1.56	1.32	1.44	0.42	4.21
Ours	1.43	1.30	1.38	0.35	4.10

**Table 3:** Experimental comparison of the proposed DDCATNet architecture (“Ours”) and its SOTA counterparts using the public Cube+ dataset [35].

Method(s)	Mean	Median	Tri-mean	Best 25%	Worst 25%
FFCC [10]	1.38	0.74	-	0.19	3.67
Bianco-CNN [45]	4.82	4.28	4.42	2.54	8.05
SqueezeNet-FC4 [12]	1.35	0.93	-	0.30	3.24
MDLCC [46]	1.24	0.83	-	0.26	2.91
C5 [18]	1.39	0.79	-	0.24	3.55
TLCC [47]	1.31	0.97	-	0.29	2.99
Autoencoder [48]	3.62	2.27	3.08	1.60	6.94
ESE [49]	1.68	1.30	1.38	0.44	6.94
HCC-RGB [50]	1.35	0.84	-	0.34	2.99
HCC-MS [50]	1.16	0.65	-	0.32	2.84
CDNet [44]	1.66	1.21	1.32	0.41	3.67
Ours	1.09	0.54	1.28	0.27	2.71

**Table 4:** Experimental comparison of the proposed DDCATNet architecture (“Ours”) and its SOTA counterparts using the LSMI [33] and Cross-Camera datasets.

Method	LSMI Device-Specific															Cross-Camera	
	Galaxy					Nikon					Sony						
	Mean	Median	Tri-Mean	Best 25%	Worst 25%	Mean	Median	Tri-Mean	Best 25%	Worst 25%	Mean	Median	Tri-Mean	Best 25%	Worst 25%	Mean	Median
Patch-CNN [39]	2.86	2.29	2.43	1.11	5.57	3.11	2.40	2.60	1.18	6.25	3.03	2.64	2.74	1.56	5.23	4.82	4.24
Angular GAN [40]	3.79	2.99	3.23	1.54	7.16	4.01	3.35	3.47	2.29	6.90	5.02	4.69	4.76	3.12	7.54	4.69	3.88
LSMI-U [33]	2.86	2.32	2.35	1.19	5.58	2.24	1.37	1.74	0.90	4.73	2.59	2.24	2.31	1.38	4.40	2.31	1.89
MIMT [43]	-	-	-	-	-	-	-	-	-	-	-	-	-	-	-	2.48	2.00
AID [51]	1.66	1.41	-	-	-	1.71	1.34	-	-	-	1.66	1.35	-	-	-	2.04	1.73
CDNet [44]	1.58	1.26	1.35	0.69	3.01	1.87	1.29	1.40	0.76	4.04	1.69	1.34	1.44	0.72	3.25	1.93	1.54
Ours	1.52	1.18	1.25	0.63	2.88	1.69	1.20	1.37	0.74	3.99	1.65	1.30	1.40	0.67	3.17	1.88	1.47

**Table 5:** Experimental comparison of the proposed DDCATNet architecture (“Ours”) and its SOTA counterparts using the LSMI [33] and Cross-Camera datasets.

Method(s)	Galaxy		Nikon		Sony		Cross-Camera	
	Single	Multi	Single	Multi	Single	Multi	Single	Multi
Patch CNN [39]	2.04	3.21	2.05	3.52	1.99	3.36	2.02	3.35
LSMI-U [33]	2.85	2.55	1.49	2.30	1.92	2.34	2.10	2.40
AID [51]	1.19	2.03	1.11	2.36	1.01	2.16	1.10	2.14
CDNet [44]	1.24	1.85	1.35	2.34	1.20	2.07	1.26	3.07
**Ours**	1.17	1.70	1.26	2.20	1.10	1.97	1.19	2.98

## Data Availability

The original contributions presented in this study are included in the article. Further inquiries can be directed to the corresponding author.
